# Pleomorphic Adenoma of External Auditory Canal: Case Report of First Endoscopic Resection and Literature Review

**DOI:** 10.3390/medicina56050248

**Published:** 2020-05-20

**Authors:** Sven Beckmann, Matthias S. Dettmer, Marco D. Caversaccio, Roland Giger, Lukas Anschuetz

**Affiliations:** 1 Department of Otorhinolaryngology, Head and Neck Surgery, Inselspital, Bern University Hospital, University of Bern, 3010 Bern, Switzerland; sven.beckmann@insel.ch (S.B.); marco.caversaccio@insel.ch (M.D.C.); lukas.anschuetz@insel.ch (L.A.); 2 Institute of Pathology, University of Bern, 3010 Bern, Switzerland; matthias.dettmer@pathology.unibe.ch

**Keywords:** Pleomorphic adenoma, external auditory canal, ceruminal gland tumor, ceruminous adenoma, endoscopic ear surgery

## Abstract

Ceruminous pleomorphic adenoma is a very rare, mostly benign tumor originating from the ceruminal glands in the external auditory canal. Histologically, it is a mixed tumor with epithelial and stromal parts of different proportions, and is recognized today by the World Health Organization (WHO) as a ceruminous adenoma. Similar to the pleomorphic adenoma of salivary glands, recurrence or malignant degeneration with cellular atypia and metastasis can occur on rare occasions. Here, we describe an 87-year old female patient with a growing spherical mass in the right external auditory canal. After exclusive endoscopic tumor resection, a ceruminous pleomorphic adenoma was histologically diagnosed. Due to the absence of nuclear pleomorphism, no increased mitotic rate, no perineural invasion and no fusion transcripts of the *MYB* or *MYBL1* gene loci, an adenoid cystic carcinoma could be excluded. The postoperative course was without any evidence of complications. A literature review identified 44 articles with 49 patients that were considered. Hearing loss and ear sensations were the most commonly reported symptoms. Most cases underwent an excision via an endaural or retroauricular approach. Recurrences were described in four patients, three of which had a malignant transformation.

## 1. Introduction

Pleomorphic adenoma is one of the most common tumors in the head and neck area. It usually occurs in the large salivary glands, although numerous other localizations have been described [1]. With an incidence of 4.3/100,000 persons per year, pleomorphic adenoma in the parotid gland is the most common salivary gland tumor; it should be resected because of its growth tendency and possible malignant transformation [2]. Therapeutically, a superficial or total parotidectomy is typically performed in most cases, although recently, partial superficial parotidectomy and extracapsular dissection as limited surgery are a suitable alternative in selected patients [3]. There is even the possibility of an endoscopically assisted extracapsular dissection with cosmetically favorable outcomes, although the long-term outcomes of this procedure are not well known [4].

A much more rarely affected area for pleomorphic adenomas is the external auditory canal (EAC), as first described in 1951 [5]. Since then, only a few cases have been reported in the literature. The manifestation of pleomorphic adenomas in the EAC is not completely understood, although the ceruminal glands may be the origin of the tumors.

With the spread of endoscopic surgery for middle ear pathologies, this technique has also been used successfully for diseases in the EAC [6]. To our knowledge, this is the first reported case of exclusive endoscopic resection of a pleomorphic adenoma of the EAC. 

## 2. Presentation of Case Report

An 87-year-old female patient underwent a superficial biopsy of a 1.2 × 0.4 cm spherical mass in the right EAC at the transition from the cartilaginous to the bony part in an external setting. The mass grew in the five subsequent months and was treated again in an external setting with a second, more extensive biopsy of the 1.3 × 1.0 × 0.6 cm actual-size lesion. 

The initial biopsy revealed a ceruminous adenoma, whereby a ceruminous pleomorphic adenoma or ceruminous adenocarcinoma could not be excluded due to the superficial nature of the biopsy. The second biopsy of the growing mass identified a basaloid biphasic neoplasia with chondromyxoid stroma. In addition to a ceruminous pleomorphic adenoma, a ceruminous adenoid cystic carcinoma was another differential diagnosis in the second biopsy.

**Figure 1:**
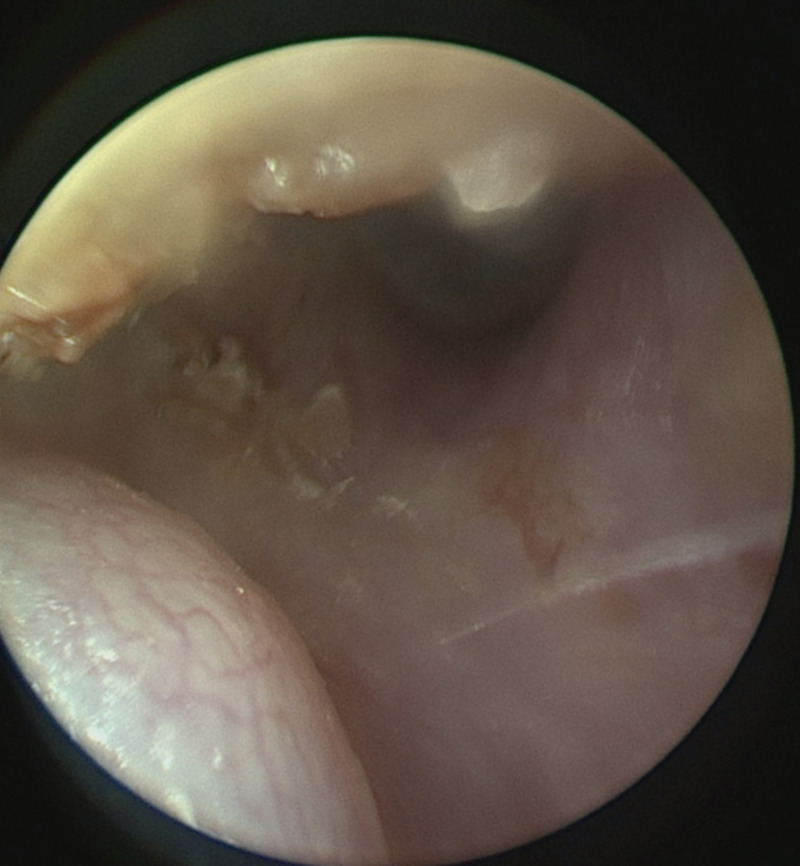
Endoscopic view on the tumor at the right posterior external auditory canal.

**Figure 2:**
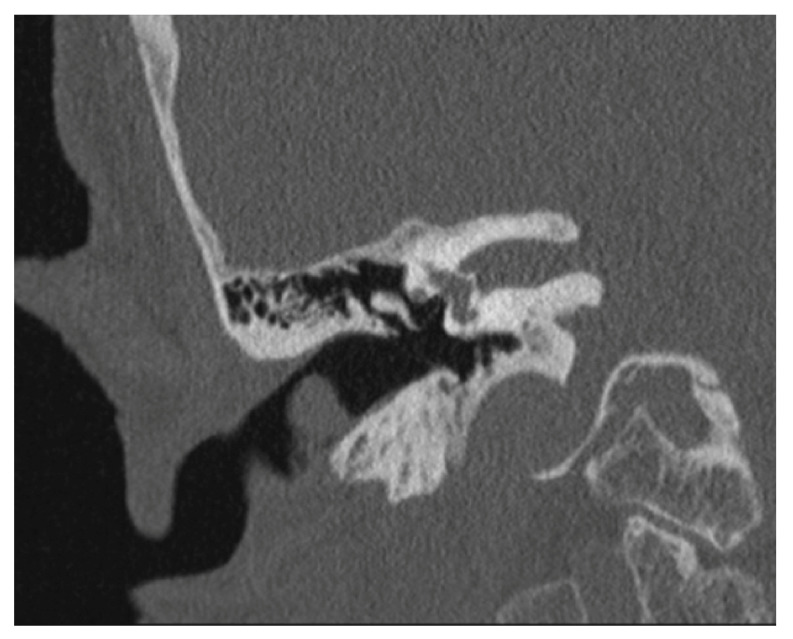
Computed tomography imaging (coronal plane) shows a homogeneous tumor in the right external auditory canal without evidence of bone erosion.

The patient was then allocated to our ENT-Department. Apart from having a known sensorineural hearing loss on both sides, she was asymptomatic. Otalgia, otorrhea, facial paralysis or taste disorders were absent. Otoendoscopy revealed a mass at the posterior wall of the EAC with otherwise normal ear findings (Figure 1). Computed tomography imaging (CT) showed a soft tissue-like exophytic mass without bony erosion of the EAC (Figure 2).

Therefore, it was decided that an exclusive endoscopic tumor resection should be performed up to the cartilaginous and bony part of the EAC, including perichondrium and periosteum, while covering the defect using a biomembrane (Bio Design, COOK Medical^®^). The tumor was resected according to its macroscopic aspect, and subsequently, resections in all directions were performed during the same operation. This comprised a complete resection.

**Figure 3:**
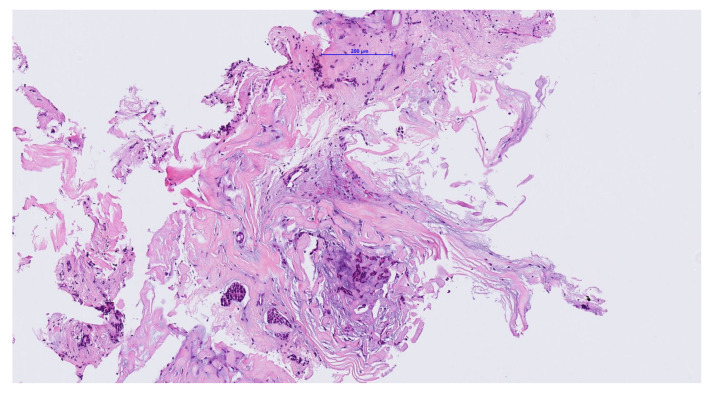
Admixture of ductules/tubules and matrix stroma including myxoid and hyaline foci. Some spindle shaped cells in loose connective tissue and fibrous backgrounds. No evidence of malignant growth pattern, no perineuralor vascular invasion. No nuclear pleomorphism or increased mitotic rate.

Finally, the existence of a ceruminous pleomorphic adenoma was assumed due to a lack of nuclear pleomorphism, no increased mitotic rate, no perineural invasion and no fusion transcripts of the *MYB* or *MYBL1* gene loci for an adenoid cystic carcinoma (Figure 3).

The postoperative course showed a completely re-epithelialized EAC at 6 weeks and without any evidence of complications or regrowth up to 4.5 months after the operation.

## 3. Discussion

The rarity of this pathology in this localization led us to conduct a literature review on current diagnostic and therapeutic management. Included were case reports of pathologically confirmed pleomorphic adenoma in the area of the EAC in English from any time period. Case reports published in other languages were excluded, as were localizations other than the EAC, as well as tumor invasion to the EAC from the parotid gland.

**Figure 4:**
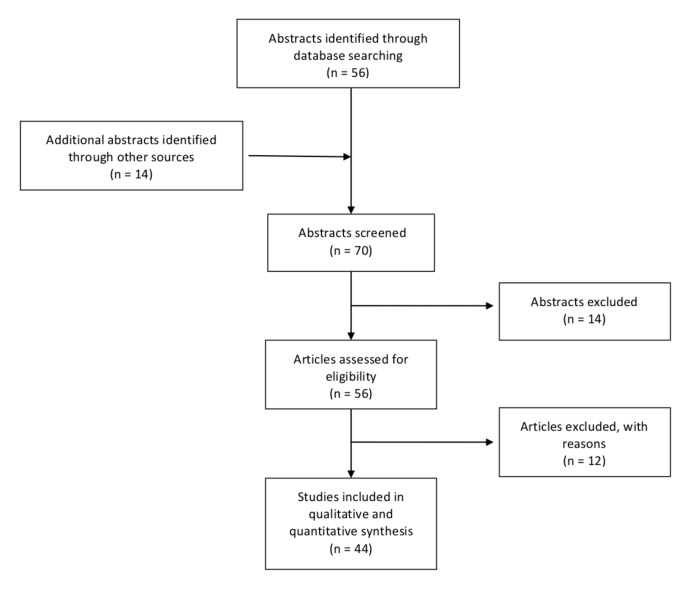
Flow diagram of case report selection.

The search and review process is illustrated in Figure 4. A total of 44 articles describing 49 patients with pleomorphic adenoma of the EAC were included. Summaries of the included patients are presented in Table 1.

Pleomorphic adenoma in the EAC usually occur in the posterior part (34.7%, n = 17), seem to be unrelated to gender and are usually symptomatic, e.g., through hearing loss or ear sensations (40.8% each, n = 20). The entity requires adequate surgical therapy with sufficient safety margins as well as regular and long-term follow-up due to the potential risk of recurrence in 8.2% (n = 4) and malignancy in 6.1% (n = 3) of cases. So far, only endaural and retroauricular approaches and one transcanal approach have been described in the literature; our case reports the first endoscopic approach, as described above. We chose endoscopic resection due to the minimal invasiveness of the procedure with short postoperative pain and healing times for the comparable cholesteatoma of the EAC [6]. Compared to the microscopic approach, the endoscopic approach makes it possible to obtain a continuous overview of the surgical area in the EAC through one-handed endoscopic handling, although this requires a one-handed surgical technique. Notably, bleeding control as a one-handed technique requires practice, but can safely be achieved by topical and injected epinephrine [49].

Tumors emerging from the ceruminal glands were first described in 1894 by Haug [50], and were previously referred to as *ceruminoma* in the literature, irrespective of their type [51]. Nowadays, ceruminous pleomorphic adenoma are recognized by the WHO as ceruminous adenoma. In total, only 2.4% of all tumors in the EAC are tumors of the ceruminal glands [52]. Squamous cell carcinomas in the area of the EAC occur much more frequently, although other tumors such as basal cell carcinoma and malignant melanoma may occur there too [53]. Immunohistochemical staining makes it possible to differentiate among these tumor pathologies, in addition to their clinical, radiological and histological properties [54]. Other benign lesions such as osteoma, exostosis, inflammatory polyps of the middle ear and EAC-cholesteatoma are further possible differential diagnoses [41].

An initial classification of the ceruminal gland tumors was proposed by Cankar and Crowley [11], and later by Welti et al. [55], into the following groups: (1) ceruminous adenoma, (2) pleomorphic adenoma, (3) ceruminous adenocarcinoma, and (4) adenoid-cystic carcinoma. This was later extended by Mansour et al. [24], as advocated today by the WHO (Table 2) [56]. After ceruminous syringocystadenoma papilliferum, ceruminous pleomorphic adenoma is the second rarest benign tumor of the ceruminal glands [30]. In addition, mixed tumors from adnexal structures in other skin areas can appear and are called chondroid syringoma [57]. The term “chondroid syringoma” can therefore be used synonymously with pleomorphic adenoma in the area of the EAC.

The histogenetical origin of pleomorphic adenoma in the EAC is controversial in the literature. In addition to an originating from ceruminal glands [22,31], an ectopic salivary gland tissue origin was discussed [19]. However, in contrast to ectopic salivary gland tissue in the middle ear [58], this evidence could never be proven for the EAC [39]. The ingrowth of a pleomorphic adenoma from the parotid gland into the EAC by the Foramen Huschke, invading the fallopian canal, cartilaginous fissures of Santorini and petrotympanic sutures are other possible routes [41,59]. Therefore, parotid gland origin should be always excluded using clinical exams and imaging studies.

The diagnosis of a pleomorphic adenoma can be confirmed by FNAC [27]. Benign and malignant forms can be distinguished using FNAC [28]. If FNAC is not diagnostic, incision biopsy is the method of choice [36]. Computed tomography imaging in these cases shows no bony erosion [36,37,44]. In magnetic resonance imaging, pleomorphic adenoma of the EAC have well-defined margins with hypointensity on T1-weighted images and hyperintensity on T2-weighted images, as in the parotid gland [29]. Due to the possible malignancy of every lesion in the EAC, excision with a complete histopathological exam should be carried out in each case [60].

In the present case, the diagnosis of a pleomorphic adenoma of the external auditory canal was delayed by the initially superficial and nonrepresentative biopsy. After a second biopsy of the growing mass, a ceruminous pleomorphic adenoma was identified, with ceruminous adenoid cystic carcinoma as the differential diagnosis. As adenoid-cystic carcinomas can feature genetic alterations in the *MYB* and *MYBL1* genes, in addition to typical perineural invasion [61,62], further molecular pathological analyses of the tissue were performed. Since no detection of fusion transcripts and no perineural invasion could be demonstrated, the diagnosis of a pleomorphic adenoma was assumed. Further development of detection methods for genetic alterations in adenoid cystic carcinoma and pleomorphic adenoma may increase the diagnostic reliability in such cases [63]. Furthermore, these genetic alterations might, in future, also serve as the basis for molecular targeted therapies in malignant cases.

Similar to the pleomorphic adenoma of the salivary glands, relapses may occur in the area of the EAC if resection is inadequate or if the tumor ruptures during surgery [41]. A local relapse 6 years after resection, which recurred within 1 year after irradiation, was described in 1967 by Batsakis [12]. The first malignant relapse with satellite nodules, cellular atypia and mitotic activity was described in 1978 by Botha et al. [16], and the first metastasizing pleomorphic adenoma of the EAC was described in 2001 by Goh et al. [28]. Even the development of an epithelial-myoepithelial carcinoma from a pleomorphic adenoma in the EAC has been described [43]. Furthermore, cases of metastatic pleomorphic adenoma without malignant transformation have been described in salivary glands [64], although there is no known occurrence of such a case in the area of the EAC so far. Therefore, we recommend complete excision according to the macroscopic aspect with subsequent resections in all directions in the same operation to ensure negative margins.

Sufficient surgical therapy with wide margins if possible should be performed with regular and long-term follow-ups [28], especially as recurrence is also possible after complete local excision. However, recurrence and malignancy rates need to be interpreted with caution, as they are widely variable and based on case reports only. Apart from the endaural and retroauricular approaches described above, our case reports the first endoscopic resection using a minimally invasive approach.

## 4. Conclusions

Pleomorphic adenoma in the EAC is the second rarest ceruminal gland tumor after syringocystadenoma papilliferum. The tumor is benign and frequently occurs at the posterior wall of the EAC. Symptoms include hearing loss or ear sensations. In addition to a thorough clinical examination, confirmation using FNAC or biopsy, and a CT to determine the extent and exclude bone erosion are recommended. The treatment of small neoplasms of the EAC should comprise excisional biopsy. Due to possible recurrence and malignant transformation, appropriate safety margins are necessary. For the same reasons, long-term follow-up is recommended.

## Figures and Tables

**Table 1:** Patient’s characteristics, type of surgery and outcome.

Author	Number/Age/Gender	EAC	Symptoms	Size	Therapy	Relapse
Mark et al., 1951 [5]	1/37/f	anterior	LM*	25 × 13 × 17 mm	Exc	No rec
Fink, 1953 [7]	1/57/m	N/A	OR	5 mm Diameter	Exc	No rec
Nandi et al., 1961 [8]	1/79/f	posterior	OL/OR/H/HL/FP/D	17 × 13 mm	Mast/En-Exc	No rec
Smith et al., 1962 [9]	1/60/f	superior	LM*/OL	35 × 10 mm	Exc/Rad	No rec
Tung et al., 1963 [10]	1/33/m	N/A	LM*/OR	15 × 8 × 9 mm	Exc	No rec
Cankar et al., 1964 [11]	1/27/f	N/A	N/A	N/A	Exc	No FU
Batsakis et al., 1967 [12]	1/71/f	posterior	N/A	20 × 20 × 15 mm	Exc/Rad	Rec
Nail et al., 1967 [13]	1/70/f	posterior	OL/OR/TI	N/A	Exc	No rec
Pahor et al., 1975 [14]	1/53/m	superior	HL/TI	25 × 20 × 15 mm	En-Exc	No rec
Baker et al., 1977 [15]	1/66/f	posterior	LM*/OR/HL	15 mm Diameter	Exc	No rec
Botha et al., 1978 [16]	1/15/f	N/A	N/A	N/A	Exc	Rec (malignant)
Goldenberg et al., 1980 [17]	1/20/f	anterior	OL/HL	N/A	S-Par	No rec
Dehner et al., 1980 [18]	1/74/m	N/A	N/A	20 mm Diameter	Exc	No FU
Chen, 1982 [19]	1/27/m	N/A	FB*/OR	10 mm Diameter	Exc	No rec
Hicks, 1983 [20]	1/44/m	N/A	F*/P*/IF	10 mm Diameter	Exc	No FU
Tanaka et al., 1984 [21]	1/52/m	posterior	OB*	10 mm Diameter	En-Exc	No rec
Collins et al., 1989 [22]	1/55/f	posterior	HL	8 mm Diameter	Re-Exc	No rec
Suzuki et al., 1991 [23]	1/51/f	anterior	OR	9 × 7 × 6 mm	Exc	No rec
Mansour et al., 1992 [24]	1/45/m	superior	HL/TI	15 mm Diameter	En-Exc	No rec
	1/52/m	anterior	TI	10 mm Diameter	En-Exc	No rec
Tang et al., 1994 [25]	1/39/m	posterior	LM*/HL	20 × 10 × 10 mm	Exc	No rec
Haraguchi et al., 1996 [26]	1/38/m	anterior	LM*	12 × 8 × 5 mm	En-Exc	No rec
Gerber et al., 1999 [27]	1/43/m	superior	OL/HL	22 × 18 × 17 mm	En-Exc	No rec
Goh et al., 2001 [28]	1/12/f	posterior	N/A	30 mm Diameter	Exc	Rec (malignant)
Masumara et al., 2003 [29]	1/62/m	N/A	LM*, F*	12 mm Diameter	En-Exc	No FU
Thompsen et al., 2004 [30]	4/N/A	N/A	N/A	N/A	N/A	N/A
Koyuncu et al., 2005 [31]	1/58/f	anterior	HL/TI/IT	16 × 8 × 4 mm	En-Exc	No rec
Kaushik et al., 2005 [32]	1/57/f	posterior	OB*/HL	11 × 10 × 10 mm	En-Exc	No rec
Karnwal et al., 2006 [33]	1/40/m	posterior	LM*	15 × 20 × 17 mm	Exc	No FU
Kuwabara et al., 2006 [34]	1/69/f	posterior	LM*	9 mm Diameter	Re-Exc	No rec
Tsukahara et al., 2006 [35]	1/36/m	posterior	HL/OL	18 × 14 × 21 mm	Re-Exc	No FU
Granell et al., 2008 [36]	1/38/m	inferior	OR/HL	17 × 11 × 8 mm	Exc	No FU
López Campos et al., 2008 [37]	1/68/m	N/A	HL	N/A	Exc	No rec
Markou et al., 2008 [38]	1/60/f	posterior	LM*/OL/TI/HL	N/A	Mast/Re-Exc	No rec
Ayers et al., 2010 [39]	1/32/f	anterior	LM*/OL	N/A	Tra-Exc	No rec
Chadha et al., 2011 [40]	1/37/f	N/A	OB*/OR/HL	N/A	Re-Exc	No rec
Kuo et al., 2011 [1]	1/N/A	N/A	N/A	N/A	Exc	No FU
Kanaan et al., 2011 [41]	1/23/f	inferior	F*/OL	10 × 7 × 7 mm	Re-Exc	No rec
Vasileiadis et al., 2011 [42]	1/34/m	posterior	LM*/HL	15 × 8 mm	Exc	No rec
Lee et al., 2012 [43]	1/40/f	N/A	N/A	N/A	Exc	Rec (malignant)
Wadhara et al., 2013 [44]	1/25/m	posterior	OB*/HL	12 mm Diameter	En-Exc	No rec
Maruyama et al., 2014 [45]	1/40/m	posterior	HL/TI	18 × 12 × 12 mm	En-/Re-Exc	No rec
Saito et al., 2014 [46]	1/40/m	posterior	FB*/HL	23 × 21 × 18 mm	Re-Exc	No rec
Jaber et al., 2015 [47]	1/55/m	N/A	OR/HL	10 mm Diameter	Re-Exc	No rec
Mohan et al., 2015 [48]	1/40/m	N/A	OL/HL	20 × 17 mm	N/A	No rec
Our case	1/87/f	posterior	A	13 × 10 × 6 mm	End-Exc	No rec

f: female; m: male; A: asymptomatic; D: dizziness; EAC: external ear canal; F*: fullness; FB*: foreign body sensation; FP: facial palsy; H: headache; HL: hearing loss; IF: infection; IT: itchiness; LM*: lump/mass; N/A: not available; OB*: obstruction/blocking; OL: otalgia; OR: otorrhea; P: pressure; TI: tinnitus; *Ear sensations (lump/mass/obstruction/blocking/fullness/foreign body sensation), Biop: Biopsy; En-Exc: Endaural excision; Exc: Excision; Mast: Mastoidectomy; Rad: Radiation; Re-Exc: Retroauricular excision; S-Par: superficial parotidectomy with tumor exstirpation parapharyngeal; Tra-Exc: transcanalic excision; End-Exc: Endoscopic excision; No FU: No follow-up: No rec: No recurrence; Rec: Recurrence.

**Table 2:** Ceruminous neoplasms of the EAC according to the WHO classification [56].

Ceruminous Neoplasms	
Benign	Malignant
Ceruminous adenoma	Ceruminous adenocarcinoma
Ceruminous pleomorphic adenoma	Ceruminous adenoid cystic carcinoma
Ceruminous syringocystadenoma papilliferum	Ceruminous mucoepidermoid carcinoma
